# Supplementary material for: The DND1–NANOS3 complex shapes the primordial germ cell transcriptome via a heptanucleotide sequence in mRNA 3′ UTRs
Source: bioRxiv. 2025 Sep 25:2025.09.25.678639. Preprint. [Version 1] doi: 10.1101/2025.09.25.678639 (PMC12485838; doi:10.1101/2025.09.25.678639)
Supplement: Supplement 1 [file media-1.pdf]

Supplementary Figure 1

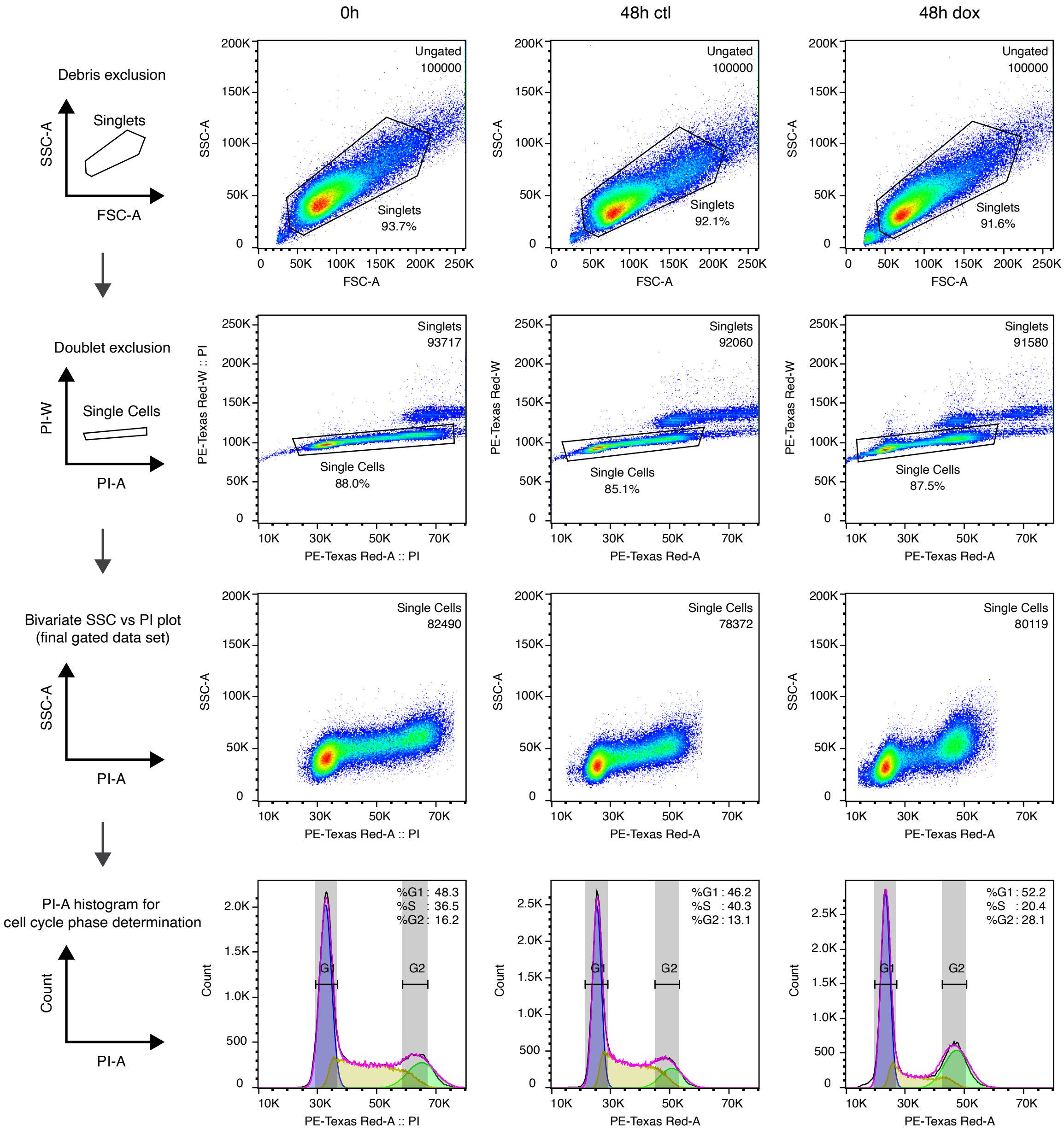

Supplementary Figure 1: Gating strategy for cell cycle analysis relate to Fig. 2b.
Representative data are shown for 0 h, 48 h control, and 48 h doxycycline-treated cells.
Fixed and stained cells were first gated on FSC-A versus SSC-A to exclude debris and on PI-A versus PI-W to exclude doublets. SSC-A versus PI-A plots with their corresponding PI-A histograms are shown as final gated dataset. DNA content histograms were used to determine the distribution of cells in G0/G1, S, and G2/M phases.
